# Vehicle–Bridge Interaction Modelling Using Precise 3D Road Surface Analysis

**DOI:** 10.3390/s24020709

**Published:** 2024-01-22

**Authors:** Maja Kreslin, Peter Češarek, Aleš Žnidarič, Darko Kokot, Jan Kalin, Rok Vezočnik

**Affiliations:** 1 Department of Structures, Slovenian National Building and Civil Engineering Institute, Dimičeva ulica 12, 1000 Ljubljana, Slovenia; ales.znidaric@zag.si (A.Ž.); darko.kokot@zag.si (D.K.); jan.kalin@zag.si (J.K.); rok.vezocnik@zag.si (R.V.); 2 Faculty of Civil and Geodetic Engineering, University of Ljubljana, Jamova cesta 2, 1000 Ljubljana, Slovenia; peter.cesarek@fgg.uni-lj.si

**Keywords:** bridge–vehicle interaction, laser scanning, road roughness, numerical modelling

## Abstract

Uneven road surfaces are the primary source of excitation in the dynamic interaction between a bridge and a vehicle and can lead to errors in bridge weigh-in-motion (B-WIM) systems. In order to correctly reproduce this interaction in a numerical model of a bridge, it is essential to know the magnitude and location of the various roadway irregularities. This paper presents a methodology for measuring the 3D road surface using static terrestrial laser scanning and a numerical model for simulating vehicle passage over a bridge with a measured road surface. This model allows the evaluation of strain responses in the time domain at any bridge location considering different parameters such as vehicle type, lateral position and speed, road surface unevenness, bridge type, etc. Since the time domain strains are crucial for B-WIM algorithms, the proposed approach facilitates the analysis of the different factors affecting the B-WIM results. The first validation of the proposed methodology was carried out on a real bridge, where extensive measurements were performed using different sensors, including measurements of the road surface, the response of the bridge when crossed by a test vehicle and the dynamic properties of the bridge and vehicle. The comparison between the simulated and measured bridge response marks a promising step towards investigating the influence of unevenness on the results of B-WIM.

## 1. Introduction

The development of weigh-in-motion (WIM) technology has led to the need for WIM systems to acquire reliable and unbiased data on all heavy-duty vehicles in free-flow traffic, including axle loads, axle spacings, gross weights and velocities. These data are crucial for many applications related to effective traffic and road infrastructure management. These include traffic studies, the pre-selection of overloaded vehicles, the design of pavements and assessment of the realistic structural safety of bridges. In general, there are two types of WIM installations: pavement WIM systems [1,2] and bridge WIM systems (B-WIM) [3]. Both measure the dynamic response of the supporting structures, pavements or bridges, and attempt to calculate the best approximation of the static axle loads and gross weights of the crossing vehicles. A key advantage of B-WIM systems compared to their pavement versions is that they can be moved from one location to another and installed and maintained without disrupting traffic or damaging the pavement. Another advantage is that B-WIM systems can collect information about the actual structural behaviour under traffic loading [4]. This information is crucial for the calibration of numerical models of bridges [5].

Instrumented bridges are sensitive to environmental influences. Therefore, the accuracy of the B-WIM weighing results depends on various factors. One of the most important is the road surface [6], which causes unpredictable dynamic vehicle–bridge interaction (VBI) [7]. The uneven road surface in the form of settlements, potholes or ruts causes deviations from a straight driving line in different wavelength ranges, which increases the dynamic interaction between the vehicle and the bridge.

The VBI depends on [8] the properties of the bridge (geometry, support conditions, stiffness, damping properties), the vehicle (axle spacing, weight distribution, stiffness and damping properties of the suspension system) and the load (velocities, load positions and number of vehicles in the loading event). Numerical models of VBI can be distinguished based on the assumed model complexity of the bridge (beams, shells, solids), the vehicle (point mass, springs and dashpots with multiple degrees of freedom) and their coupling (point contact, surface contact); see [9,10] and the references therein for a comparison of different approaches to VBI modelling. The unevenness of the road surface is mainly generated numerically in the VBI literature [9,11,12,13].

In recent years, the technological readiness of the various systems for measuring road surface unevenness has increased significantly. As a result, 3D coordinate measuring sensor systems have become standard tools for this increasingly automated activity. In contrast to the traditional line-based measuring systems [14], a near-surface metric description of the pavement geometry can be captured using various image -or laser-based static or mobile measuring systems [15,16,17]. One such surface technology that can efficiently scan all visible bridge features is static terrestrial laser scanning (TLS), described in Section 2.1. These survey methods have been used for more than a decade in various applications, including precise monitoring of deformations [18], and were used for the metric survey of the test case presented in this paper. Static terrestrial laser scanning technology can capture road surface irregularities with sub-centimetre accuracy and sufficient spatial resolution for modelling vehicle–bridge interaction.

The main objective of the research presented here is to utilise precisely measured 3D road surface models in the numerical modelling of VBI. Firstly, a methodology for accurately measuring the 3D road surface is presented, followed by the development of a mathematical model in the Abaqus software to simulate a vehicle crossing over a bridge with an uneven road surface. Although this model was originally developed for a selected bridge and vehicle, it is adaptable to different vehicle types and allows the evaluation of time domain strain responses at any bridge location under different conditions, including different vehicle types, lateral positions and speeds, road surface unevenness and bridge types. Since the time domain strains are fundamental components of the B-WIM algorithms, the proposed approach facilitates the analysis of the different factors affecting the B-WIM results. The methodology was tested on a real bridge on which extensive measurements were performed, including measurements of the road surface and the dynamic properties of the bridge and the vehicle. The strain response of the bridge superstructure was measured while selected vehicles passed over the bridge. Finally, the strains from the numerical model were compared with the measured strains.

## 2. Methodologies

This section describes the methodology for using measured road surface unevenness in the numerical modelling of VBI. Section 2.1 introduces the operating principle of the TLS, followed by a brief and concise overview of the main steps to create 3D surface models. The latter represent the input for the road surface model for VBI. Section 2.2 then explains the entire model for simulating the vehicle crossing a bridge with an uneven surface.

### 2.1. Road Surface Modelling

Various surveying methods can provide a metric surface 3D model of the road surface (e.g., laser scanning, photogrammetry, motorised tacheometry, etc.). All of these remote sensing methods are efficient and can capture the road geometry without interfering with traffic. In this work, static terrestrial laser scanning (TLS) [19] was used as it provides the final result with sufficient accuracy and adequate surface detail.

**Figure 1:**
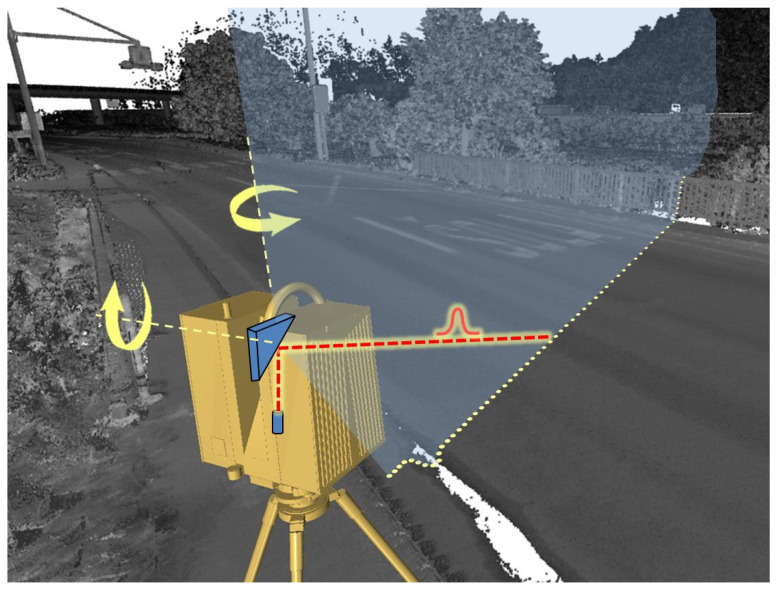
The common operating principle of static TLS.

In principle, TLS is based on direct measurements of the polar coordinates (i.e., the horizontal and vertical angle and distance) of spatial objects, reached using short laser pulses transmitted in small incremental angular steps. The result is a very dense set of individual 3D points, commonly referred to as a point cloud. Such a dataset provides information about the geometry of the object, including dimensions, inclinations and unevenness, which can be retrieved from such a 3D snapshot at any time. The accuracy of the points decreases with the distance from the scanner, but can reach the millimetre range at typical working distances. The internal geometry of a static TLS point cloud is generally superior to a moving platform-based scan (from a vehicle or drone) in terms of its accuracy and spatial resolution (i.e., point density). The technical parameters of individual TLS instruments are diverse and can vary depending on the maximum range, spatial resolution, accuracy, acquisition speed, etc. An example of the technical documentation on the instrument used in this study can be found in [20]. Figure 1 shows the usual operating principle of TLS, in which the distance is calculated by measuring the time of travel of the individual laser pulses.

In many applications, the captured point clouds cannot be used directly. The individual points do not contain any information about the type of target (they do not contain any structure or classification). Therefore, point clouds may require additional processing to obtain the final results, such as surface models [21]. These processing steps are becoming increasingly automated, but still require some human intervention when deciding on the quality measures, minimum details or data size. In general, the following processing steps can be distinguished for point clouds:

- Positioning within the reference coordinate system,
- Filtering of the point cloud,
- Point-to-surface transformation,
- Surface optimisation (e.g., decimation and smoothing).

In static TLS, the first step is performed using fixed natural or artificial targets whose coordinates are available in the reference coordinate system. This step is much more complex if the measurement platform is moving and an additional specialised data positioning unit is required.

Filtering or removing point clouds is a step that has generally not yet been fully automated. The main objective is to mostly semi-automatically remove all points that do not belong to the area of interest.

**Figure 2:**
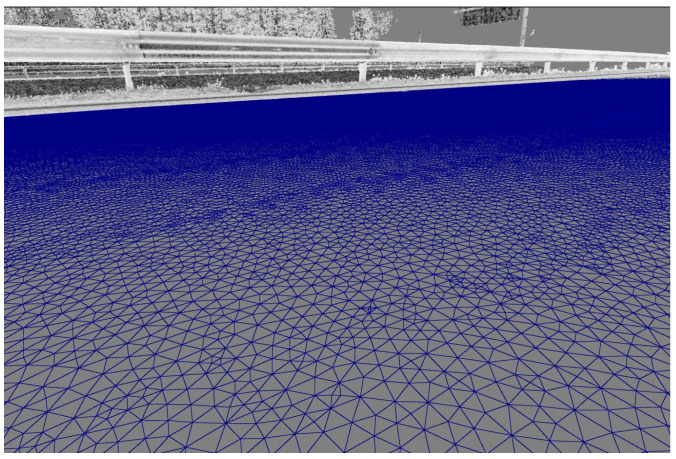
A surface model composed of irregular triangles.

In the third step, the point model is converted into a surface model by establishing the connections between the individual points. There are different surface models, of which regular (e.g., gridded) and irregular (e.g., triangular irregular network, TIN) models are most commonly used for the representation of road surfaces. Figure 2 shows an example of a triangulated irregular surface model.

In the final step, the surface model is further optimised to meet the data size and level of detail requirements. In the context of this research, the main objective of this step was to reduce the impact of the instrument’s inherent inaccuracy (measurement noise) on the quality of the final surface model. For this task, different automatic filtering methods such as the Gaussian low-pass filter [22] can provide a good compromise between a reduction in and the preservation of characteristic details (unevenness). In addition, data reduction can also be helpful to adjust the model size while preserving the overall shape of the road.

### 2.2. Modelling the Vehicle–Bridge Interaction Considering the Measured Road Surface Unevenness

**Figure 3:**
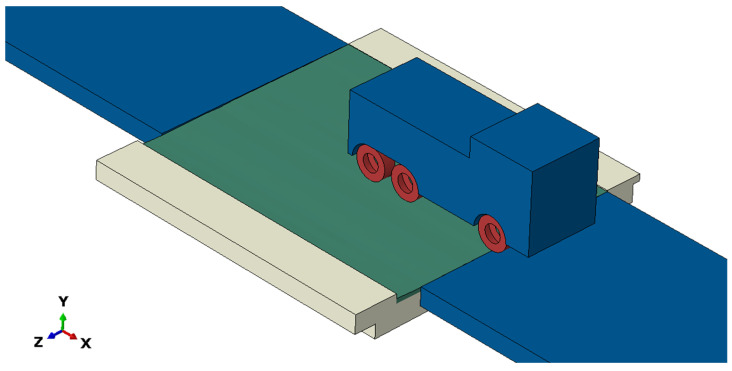
Coupled model for vehicle–bridge interaction considering the measured unevenness of the road surface.

A measured roadway profile, determined using the procedure presented in Section 2.1, is an important input for the numerical model of a vehicle crossing a bridge. The necessary components of the model are models of (i) a vehicle, (ii) a bridge with a road surface, including the carriageway before and after the bridge, and (iii) the vehicle–bridge interaction. We chose the Abaqus software [23] to create the complete model of the VBI (Figure 3).

**Figure 4:**
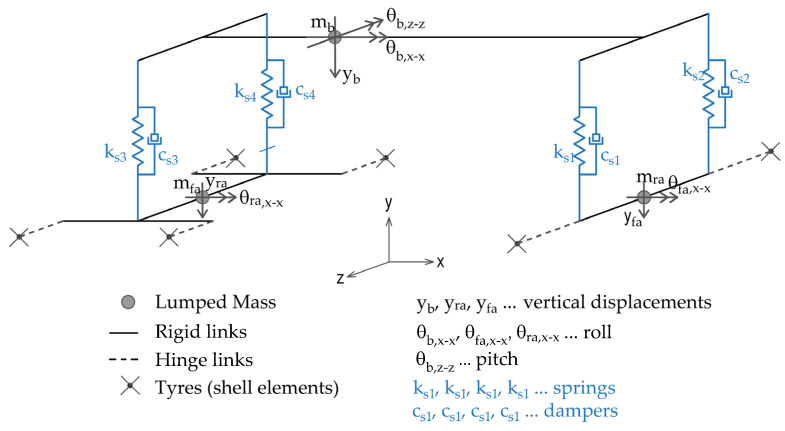
Numerical model of the vehicle.

The model represents the vehicle using a multibody system, as proposed in the literature [12,24,25]. It consists of three lumped masses representing the vehicle body, the front axle and the rear axles. It has eight degrees of freedom: translation in the driving direction, a vertical translation for each of the three masses, roll and pitch rotations of the body mass and two roll rotations for the front and rear axles (Figure 4). The masses are connected in the vertical direction with springs and dampers, which represent the suspension of the vehicle, and with rigid link elements, which constrain displacements in the direction of driving. Translations in the transverse direction and the torsional rotations and pitching rotations of the axles are restrained.

The bridge structure and the road surface of the bridge, with particular attention paid to the unevenness of the road surface, are modelled as 3D solids and discretised using 3D solid finite elements. The triangular or gridded surface model presented in Section 2.1 is extended by the thickness of the pavement layer to create a 3D solid part. In our model, both parts were created in a selected CAD program, exported as an ASCII (.igs) file and then imported into Abaqus CAE.

The bridge and vehicle interact via tyres. In the literature on vehicle–bridge interaction, tyres are commonly modelled with elastic springs connected to the axles. A further improvement in the tyre model consists of using multiple springs, in the longitudinal, lateral and radial directions. This allows the tyre–road contact to be treated according to a force law specified for the contact area. Another alternative is to model the tyres as rigid bodies which are connected to the axles with springs at their centre of rotation. A force law for the contact between the rigid tyre surface and the road surface can then be enforced via a penalty formulation, augmented Lagrangian method, etc. In the present numerical model, which considers a measured uneven road surface, numerical difficulties arise when a vehicle with tyres modelled as springs or rigid bodies encounters a hole or a bump in the road. In such cases, the time steps become very small, which increases the calculation time or can lead to a divergence in the time integration. These problems can be avoided by modelling the tyres as elastic bodies with known geometric and material properties and a known inflation pressure. In this way, hard contact is mitigated by deformable tyres, as is the case in reality. In the present model, we have adopted the approach presented in [26,27], where the tyres are modelled as 3D shells with additional simplifications. Firstly, we do not model the tyre rim, but constrain the tyre bead to its centre, which is located on the axle of the vehicle, and secondly, we model the tyres as 3D shells with homogeneous and isotropic material properties. This choice was made because we do not focus on tyre stresses; rather, our goal is to represent the tyre as an elastic body in contact with the road surface according to a realistic contact area. To simulate the interaction between the tyre and the road surface, our model uses a surface-to-surface contact formulation, employing Abaqus’ default penalty formulation to resolve the contact stresses.

The model presented allows the calculation of the bridge’s response to vehicles crossing the bridge at different velocities and lateral positions. The simulations of these crossings are performed using an implicit dynamic analysis.

## 3. Field Measurements

Extensive measurements were carried out on a real bridge to collect the essential data required to create and verify the VBI model. These measurements included assessments of the unevenness of the road surface, measurements of the dynamic properties of the bridge and the vehicle and evaluations of the bridge’s response during the passage of a known vehicle.

### 3.1. Test Bridge Geometry

**Figure 5:**
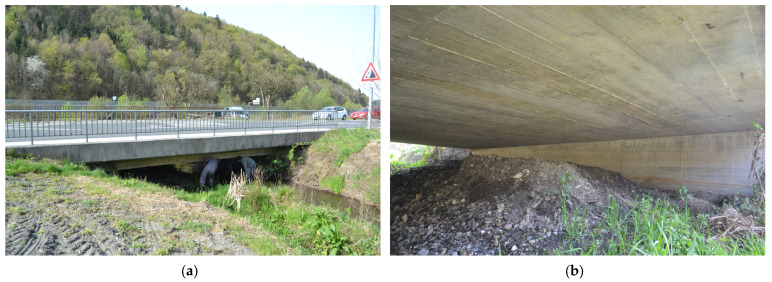
Test bridge. (**a**) Side view; (**b**) under the bridge.

**Figure 6:**
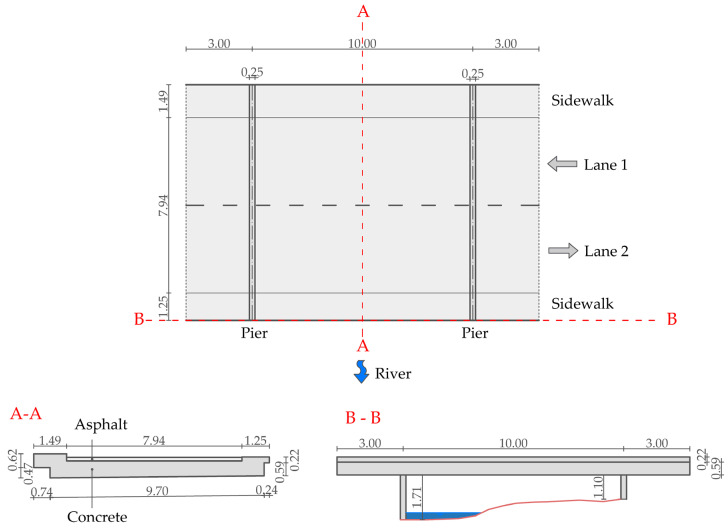
Dimensions of the test bridge, measured in the field (plan view and cross sections).

The test case is a 16 m long reinforced concrete bridge. Its structural system consists of a slab and two piers. Selected views of the bridge are shown in Figure 5. The bridge was designed in 1945 and later partially reconstructed, but the details of the reconstruction were not found. Therefore, the dimensions of the bridge were determined using on-site measurements. The plan view and cross-sections are shown in Figure 6.

**Figure 7:**
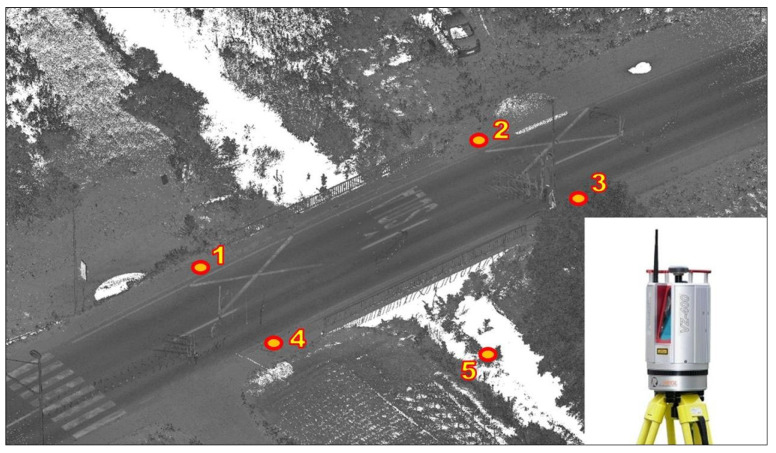
RIEGL VZ-400 scanner and survey station locations (red and yellow dots).

The geometry of the test bridge was determined using a TLS survey from five spatially separated locations (see Figure 7). Datasets from four locations along the road were used to create the final model of the road surface (locations 1–4). None of these sites were located on the bridge to avoid the negative effects of vibration on the data quality. Scanning at the fifth location under the bridge (location 5) provided additional information and the dimensions of the underlying bridge structure details. These data were needed in the fine-tuning phase of the numerical simulation tests.

The RIEGL VZ-400 [20] static laser scanner was used for the survey. At each scan location, a point density of approximately 5 mm (i.e., 5 mm at a distance of 10 m) was selected for the vertical and horizontal directions. The point clouds were later transformed into a preselected local coordinate system using retroreflective artificial targets.

### 3.2. Dynamic Properties of the Bridge and Vehicle

**Figure 8:**
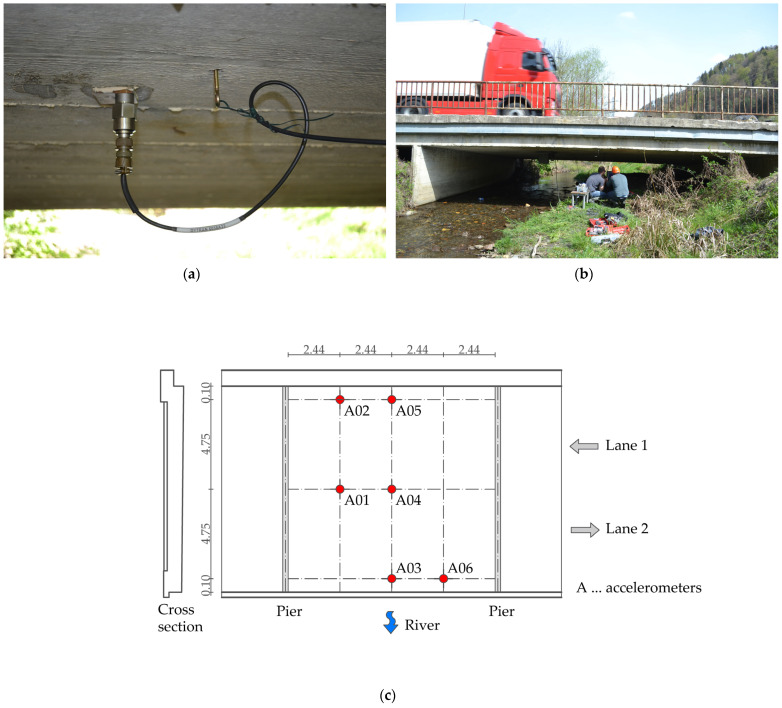
Measurements of the bridge’s dynamic properties: (**a**,**b**) instrumented accelerometer and data acquisition; (**c**) locations of the accelerometers at the bottom part of the slab.

**Figure 9:**
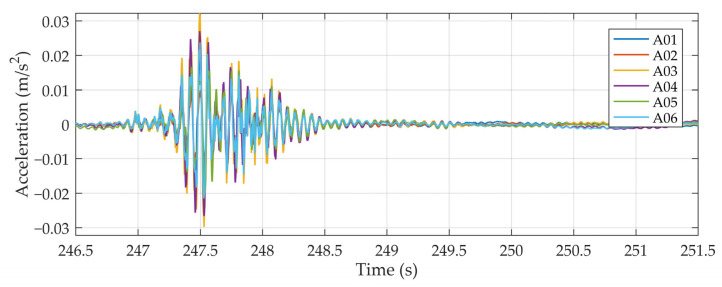
Bridge acceleration response to a random vehicle.

The dynamic bridge properties, eigenfrequencies and eigenmodes were measured using six accelerometers on the bottom side of the slab. The locations of the sensors were selected to capture the expected mode shapes (Figure 8). The accelerations were recorded at 4096 data points per second. Approximately 4500 s of valuable signals was recorded while the bridge was under live traffic. Figure 9 shows an example of a 6 s acceleration response of the bridge under a crossing vehicle. The procedure for determining the dynamic properties of the bridge from a measured signal is described in Section 4.2.1.

**Figure 10:**
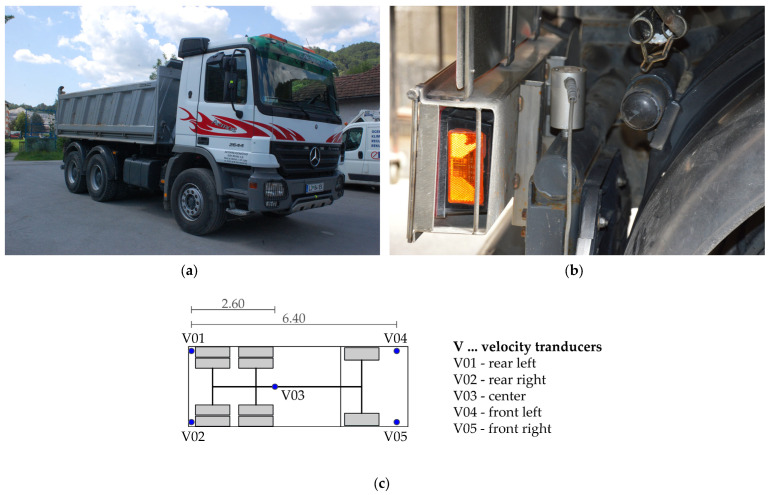
Measurements of the dynamic properties of the vehicle: (**a**) 3-axle truck; (**b**) instrumented transducers; (**c**) schematic locations of the transducers on the vehicle.

A 3-axle truck with known geometric characteristics was used as the test vehicle (Figure 10). The individual axles were weighted statically. The gross weight of the vehicle was 23.9 tonnes, with the individual axles loaded with 3367 kg, 4277 kg and 4283 kg for the front and rear axles, respectively. The stiffness of the front and rear suspension was calculated as the coefficient between the change in weight (∆F) and the change in vertical displacement (∆u) at the suspension positions caused by the difference in the axle loads of a full and an empty truck.

**Figure 11:**
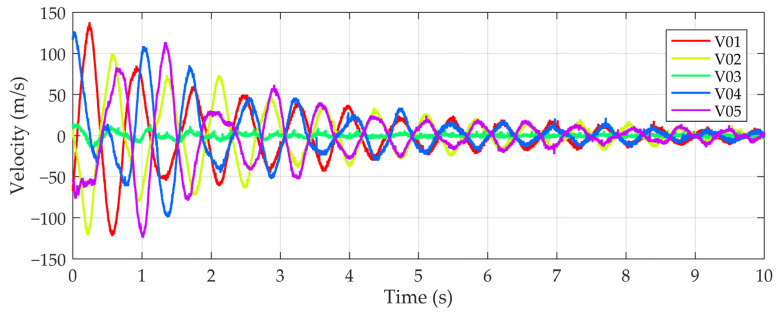
Vehicle response to a manual excitation of the vibration around the longitudinal axis.

The dynamic properties of the test vehicle in the form of eigenfrequencies and eigenmodes were measured using five velocity transducers (Figure 10). The response over time was recorded at a sampling frequency of 512 Hz. The signals were recorded while the vehicle was driving and during manual vibration excitation around the longitudinal axis (roll). An example of a 6 s signal resulting from such manual excitation of the vehicle is shown in Figure 11. The procedure for determining the dynamic properties from the measured signal is described in Section 4.2.2.

### 3.3. Bridge Response to the Crossing Vehicle

**Figure 12:**
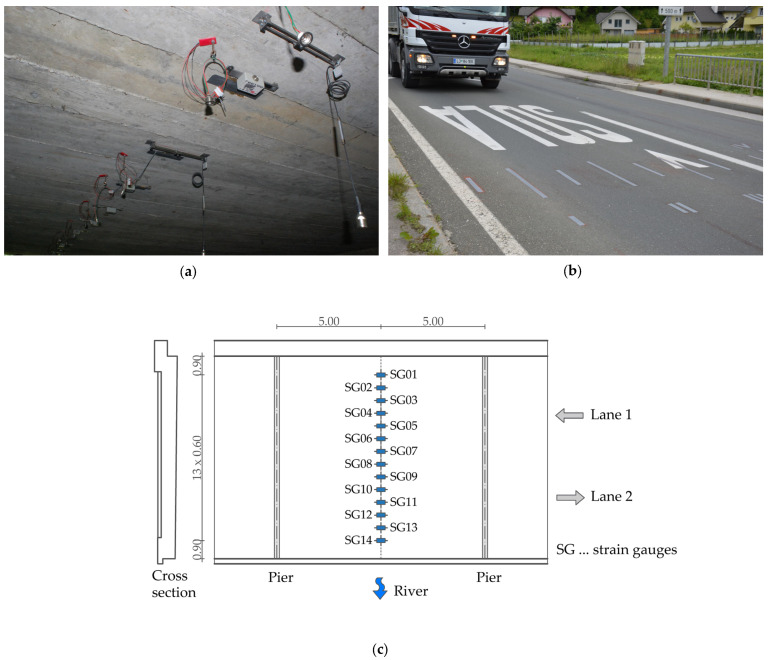
Measurements of the bridge response to crossing vehicle: (**a**) instrumented bridge; (**b**) vehicle crossing; (**c**) strain gauge locations on the bottom part of the slab.

**Figure 13:**
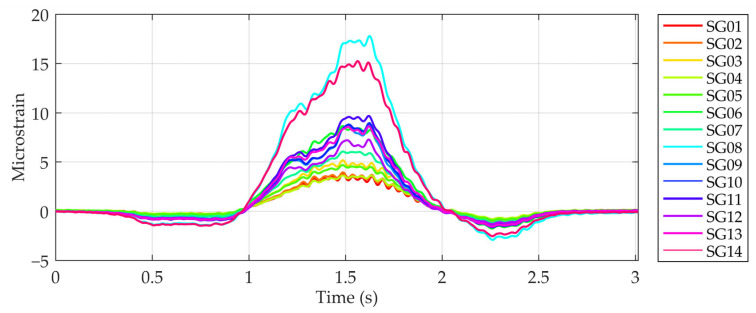
Response of the bridge in microstrains in the time domain resulting from the vehicle crossing in lane 2 at a 40 km/h speed.

The comprehensive model, which incorporates the interaction between the bridge, the vehicle and the unevenness of the road surface, was validated with measurements of the bridge’s response to crossing vehicles. The bridge was equipped with 14 strain gauges on the underside of the slab, the locations of which are shown in Figure 12. The measurements resulted in the time domain strains, recorded at 512 Hz for each vehicle crossing. A vehicle crossed the bridge in lane 2 at a speed of 40 km/h, with four crossings recorded. The bridge was closed to other traffic during these measurements to ensure accurate data collection. An example of the strain response of a selected bridge in the time domain is shown in Figure 13.

## 4. Results and Analyses

### 4.1. TLS Measurements and Verification

**Figure 14:**
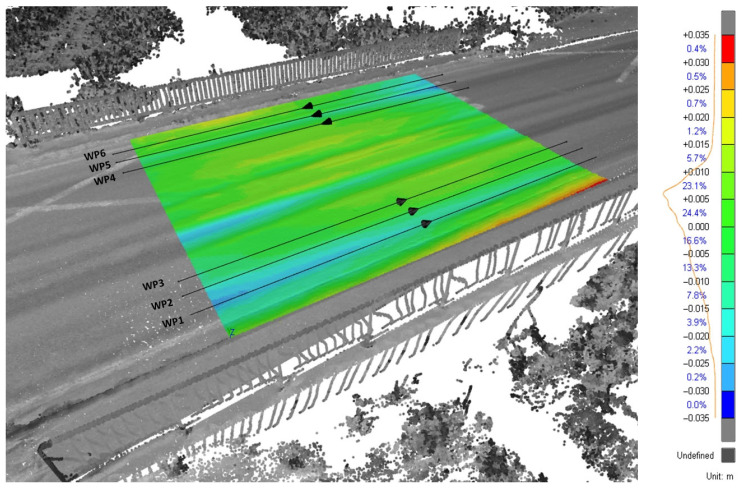
Spatial distribution of road surface unevenness.

After removing points outside the road surface, the remaining point cloud contained about half a million points. These were used as the input for the final step of converting the point model into a surface model using a triangular irregular network (TIN). Before the TIN model was used in numerical simulations, its internal geometry was compared with a best-fit planar model (minimising the deviations between the actual TIN and planar model) to obtain information on the magnitude and location of the bridge surface irregularities. This test showed that the unevenness of the surface (above and below the plane) was limited to ±3 cm. The spatial distribution of deviations from an ideal plane in Figure 14 shows the visible vehicle tracks, which indicate the pavement defects caused by heavy traffic. The legend in Figure 14 contains the deviation intervals and the percentage of deviations within each interval.

**Figure 15:**
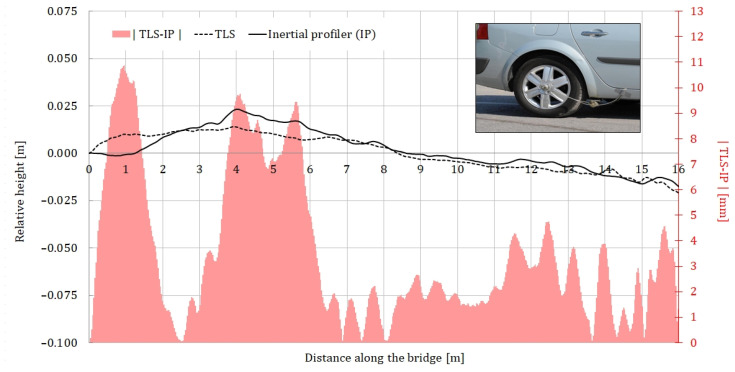
Comparison of road profiles from TLS and inertial profiler in WP1.

To verify the TLS surface model, six different longitudinal profiles of the same road section were measured using a mobile inertial profilometer, three on each lane (Figure 14, WP1–WP6). The profilometer, which can be seen in Figure 15, uses a variable-angle differential transformer and an accelerometer to determine the longitudinal profiles [28]. Each profile was measured four times, with the final longitudinal profiles representing the average of all four runs. As an example, Figure 15 compares the profiles, measured using TLS and the inertial profilometer (IP) in WP1, together with the absolute values of the differences between the two profiles.

Most of the differences between the two measurements systems are less than 4.5 mm, which is a good result considering that the accuracy of the scanner on the test site was 5 mm. This comparison leads to the following conclusions

- The general trend in road surface unevenness indicates similar results for TLS and the IP.
- For complex road surface irregularities, it is important to have complete surface data rather than individual longitudinal profiles (even if they are very accurate) for the simulation of vehicle–bridge interaction.

### 4.2. Verification of the Numerical Model for VBI Considering Road Surface Unevenness

#### 4.2.1. Bridge Model

**Figure 16:**
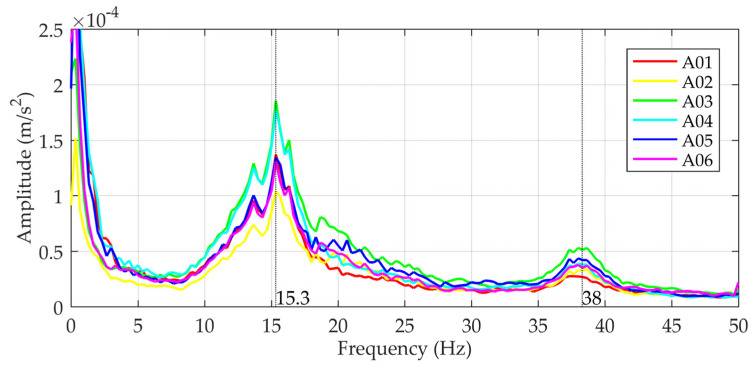
Dynamic responses of the test bridge in the frequency domain.

**Figure 17:**
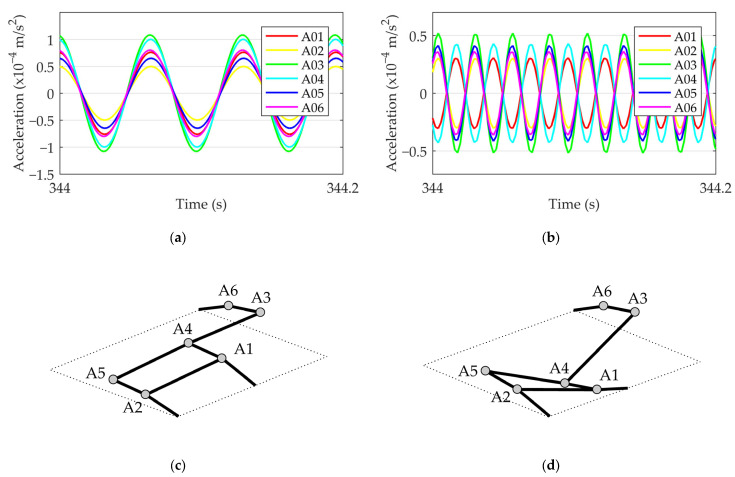
Measured eigenmodes of the bridge: (**a**,**c**) first mode; (**b**,**d**) third mode.

The dynamic properties of the structure (eigenfrequencies and damping) were measured to verify the bridge model. A fast Fourier transformation (FFT) of the measured acceleration time histories was carried out to determine the eigenfrequencies. To eliminate the influence of the vehicle weight on the dynamic response of the vehicle–bridge system, only 3 s intervals after the vehicle left the bridge were considered in the analyses. Figure 16 shows the mean frequency spectra of 44 responses. The identified frequencies were 15.3 Hz and 38.0 Hz; the corresponding damping ratios were 5% and 3%. The mode shape data of the identified frequencies were obtained by band-pass-filtering the original signal at the identified frequencies. The filtered signal with a frequency of 15.3 Hz shows that all the measurement points oscillate in the same direction at different amplitudes (Figure 17). In the case of a filtered signal with a 38.0 Hz frequency, the measuring points in the middle of the bridge (A01 and A04) oscillate in the same direction and the others in the opposite direction (Figure 17).

The eigenfrequencies of the numerical bridge model, described in Section 2.1, were calculated using modal analysis in the Abaqus software [23]. The initial material properties, the modulus of elasticity (E) and Poisson’s ratio (ν), were assumed to be E = 31 GPa and ν = 0.2 for concrete and E = 5 GPa and ν = 0.2 for asphalt. The first three are 18.8 Hz, 30.9 Hz and 45.4 Hz. Comparison of the numerical and experimental results shows that only the first and third eigenmodes were excited by the free flow of traffic (Table 1).

#### 4.2.2. Vehicle Model

**Figure 18:**
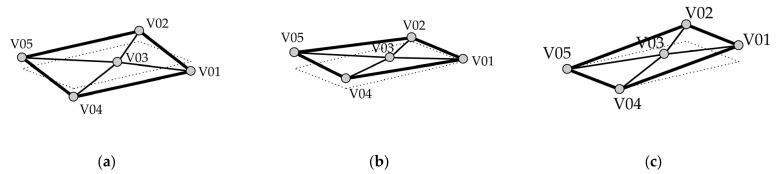
Measured eigenmodes of the vehicle: (**a**) first mode—rotation around longitudinal axes; (**b**) second mode—front axle inclination; (**c**) third mode—rear axle inclination.

The vehicle model was validated by comparing its eigenfrequencies with the measured frequencies of the actual vehicle. The eigenfrequencies of the vehicle of 1.30 Hz, 1.40 Hz to 1.50 Hz and 2.90 Hz were determined using FFT analyses of the measured velocity time histories for the first three vibration modes. Band-pass filtering of the original signals at these frequencies revealed that the first eigenmode corresponded to the rotation of the vehicle about the longitudinal axis (roll), the second to the inclination of the front axle and the third to the inclination of the rear axle (Figure 18).

**Figure 19:**
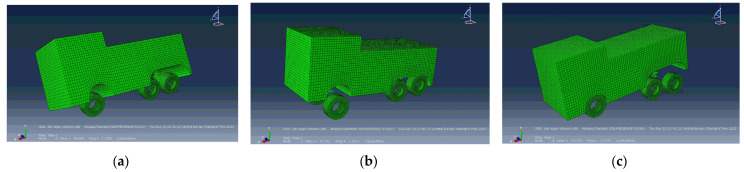
Calculated eigenmodes of the vehicle using Abaqus: (**a**) first mode—rotation around longitudinal axes; (**b**) second mode—front axle inclination; (**c**) third mode—rear axle inclination.

The eigenfrequencies of the numerical model of the vehicle described in Section 2.2 were calculated using modal analysis with the Abaqus software (Figure 19). The calculated frequencies of the first three eigenmodes were 1.20 Hz, 1.40 Hz and 2.40 Hz and agreed well with the measured values (Table 2).

#### 4.2.3. VBI Model

**Figure 20:**
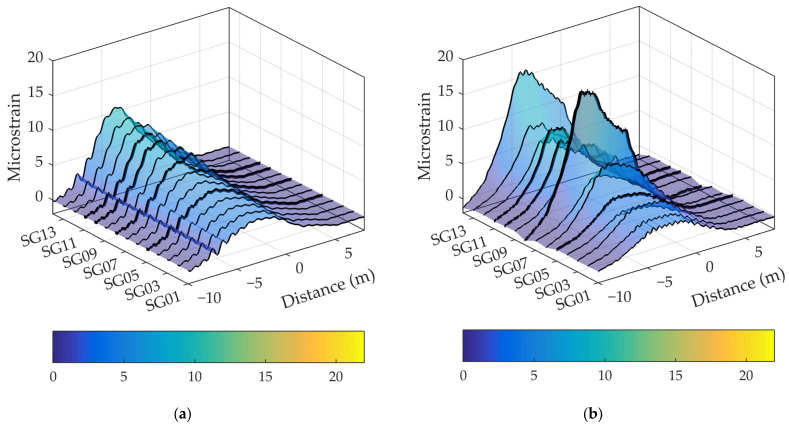
Modelled (**a**) and measured (**b**) strains for 14 locations.

**Figure 21:**
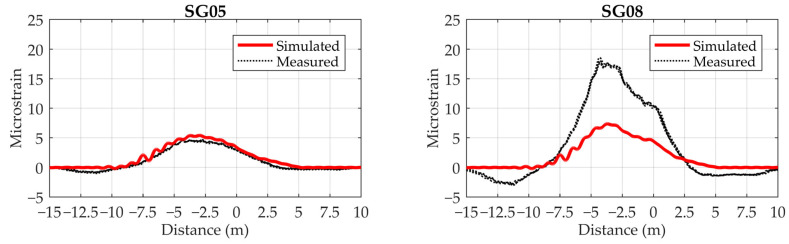

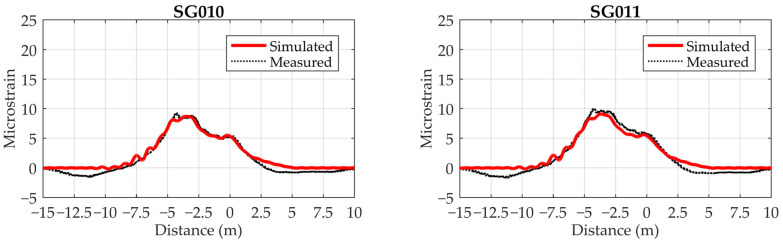
Comparison of experimental and simulated results for selected locations (SG05, SG08, SG10 and SG11).

**Figure 22:**
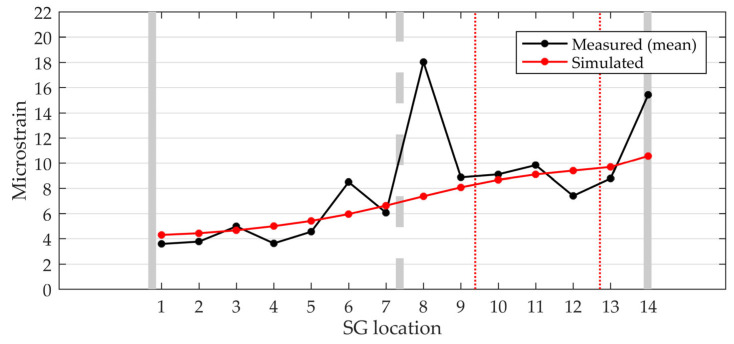
Transverse distribution of maximum strains obtained using measures and simulation.

The response of the bridge was measured during vehicle crossings to verify the proposed model of vehicle–bridge interaction (Section 2.2). Figure 20, Figure 21 and Figure 22 show the measured and simulated bridge strain responses when crossing lane 2 at 40 km/h. The mean values of four vehicle crossings in the spatial domain are shown, with the coordinate origin placed at the midpoint of the bridge. The simulated values were calculated using the numerical model described in Section 2. Figure 20 summarises the results for all 14 sensors, while Figure 21 shows detailed results for four selected locations.

**Figure 23:**
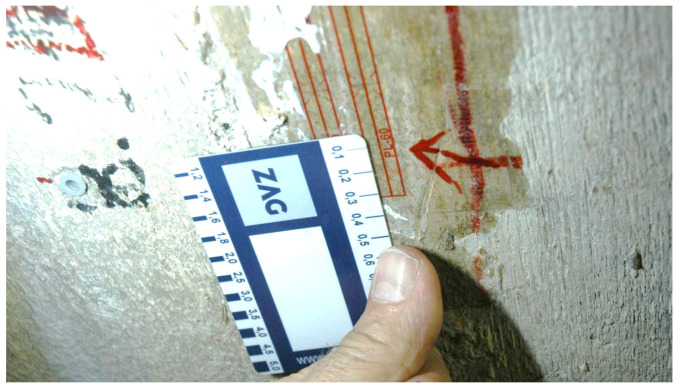
0.1 mm wide crack under the strain gauge SG8.

The simulated strains corresponded well with the measured values. In both cases, higher strains occurred under the crossing vehicle in lane 2. Significant deviations between the measured and simulated strains were only observed at locations SG8 and SG14 (Figure 20), where the measured values exceeded the simulated values by 59% and 31%, respectively (Table 3). Sensor SG8 was found to be glued over a 0.1 mm wide crack, as shown in Figure 23. We assumed that a visually undetectable microcrack was also present under sensor SG14. Therefore, we skipped the results of sensors SG8 and SG14 in the following comparisons.

For the remaining sensors, the maximum measured strains were between 3.6 µm/m and 9.8 µm/m, while the calculated values were between 4.3 µm/m and 9.7 µm/m (Table 3). The mean values of the maximum strains were 6.6 µm/m and 6.8 µm/m for the measured and calculated results, respectively.

As expected, the measured strains were negative before the vehicle entered and after it left the main span (Figure 21). The modelled results were zero only because the considered numerical model of the bridge did not encounter the overhanging part of the deck in the driving direction. The bridge model considered the overhanging effect with rotational springs on both sides of the main span.

## 5. Conclusions

This paper proposes a methodology for measuring a road surface using the precise 3D technology of terrestrial laser scanning (TLS) and applying the measured surface to a numerical model of vehicle–bridge interaction (VBI) to simulate vehicle passage over a bridge.

The model used in this study consisted of coupled sub-models of a bridge with a rough road surface and a vehicle. The triangulated network surface model of the pavement derived from laser scanning was extended to 3D so that the pavement and the bridge were modelled with 3D solid elements. The vehicle was represented as a multi-body system consisting of masses connected by springs and dampers, and the vehicle tyres were represented as elastic bodies with shell elements.

The individual sub-models (bridge and vehicle) and the coupled numerical models were verified using field measurements. The TLS road surface model agreed well with the inertial profiler, which is the recognised method for measuring the road profile. The preliminary results confirm that a numerical model of VBI that considers the measured road surface profile can realistically describe the response of the bridge in rolling traffic.

The most important result of the numerical modelling of the VBI considering the measured road surface unevenness is the realistic dynamic strain response of the test bridge under the crossing vehicles in the time domain. The results presented here show a feasible way to analyse the influence of unevenness on the results of bridge weigh-in-motion (B-WIM) in detail.

In the future, the parameters for TLS data processing need to be fine-tuned to achieve the optimal level of surface level and minimise the data size without compromising the quality of the simulation results. In addition, the numerous parameters in this complex model require a parametric analysis to investigate their influence on the results of the VBI model.

## Figures and Tables

**Table 1:** Eigenfrequencies of the test bridge.

Mode	Measurements	Numerical Model
1	15.3 Hz	15.8 Hz
2	/ ^1^	30.9 Hz
3	38.0 Hz	45.4 Hz

^1^ Not excited with traffic.

**Table 2:** Eigenfrequencies of the vehicle.

Mode	Measurements	Numerical Model
1	1.30 Hz	1.20 Hz
2	1.40–1.50 Hz	1.40 Hz
3	2.90 Hz	2.40 Hz

**Table 3:** Maximum microstrains for each location obtained using measurement and simulation.

Location	Measured Microstrain (Mean)	Simulated Microstrain	Error (%)
SG1	3.6	4.3	20%
SG2	3.8	4.4	18%
SG3	5.0	4.7	−6%
SG4	3.6	5.0	38%
SG5	4.5	5.4	19%
SG6	8.5	5.9	−30%
SG7	6.1	6.6	9%
SG8	18.0	7.4	−59%
SG9	8.9	8.1	−9%
SG10	9.1	8.7	−5%
SG11	9.8	9.1	−7%
SG12	7.4	9.4	27%
SG13	8.8	9.7	10%
SG14	15.4	10.6	−31%

## Data Availability

Data are contained within the article.
